# Supplementary material for: Bumblebee visual allometry results in locally improved resolution and globally improved sensitivity
Source: eLife. 2019 Feb 26;8:e40613. doi: 10.7554/eLife.40613 (PMC6391067; doi:10.7554/eLife.40613)
Supplement: Supplementary file 1. [file elife-40613-supp1.docx]

**Supplemental tables**

Table S1: Parameters for the power functions calculated for bumblebees in this study. Note that non-significant correlations are underlined here and plotted with dotted lines in the relevant figures.

| Relationship | X unit | Figure | Initial growth index (a) | Scaling exponent (d) | R^2^ | *P* value |
| --- | --- | --- | --- | --- | --- | --- |
| √Eye area | ITW | 2A | 0.8 | 0.45 | 0.91 | 0.003 |
| ∛EV | ITW | 2A | 0.4 | 0.45 | 0.95 | 0.001 |
| Facet number | ∛EV | 2B | 6587.1 | 0.61 | 0.78 | 0.019 |
| Complete CP | ∛EV | 2C | 5.5 | 0.16 | 0.63 | 0.059 |
| Individual CP | ∛EV | 2C | 3.5 | 0.38 | 0.86 | 0.008 |
| Binocular CP | ∛EV | 2C | 1.7 | 1.76 | 0.87 | 0.007 |
| IF angle (*ΔΦ)* | ∛EV | 3A | 1.7 | -0.09 | 0.21 | 0.367 |
| Facet diameter (*D*) | ∛EV | 3C | 28.5 | 0.71 | 0.99 | <0.001 |
| Retina thickness | ∛EV | 5A | 301.5 | 1.41 | 0.95 | 0.001 |
| Radius of curvature (*R*) | ∛EV | S1A | 1117.0 | 0.74 | 0.88 | 0.006 |
| Lens thickness | ∛EV | S2A | 71.8 | 1.17 | 0.94 | 0.001 |
| CC thickness | ∛EV | S3A | 60.4 | 0.43 | 0.32 | 0.247 |
| Eye parameter (*P*) | ∛EV | S4 | 0.8 | 0.60 | 0.90 | 0.004 |

Table S2: Topological differences between reported IO angle and our calculated IF angles for honeybees. We obtained the IO angles measured by Seidl (1982) from Fig. 2.2 in Giger (1996).

| Direction | IO angle^1^ (°) | IF angle (°) | Differences^2^ (%) |
| --- | --- | --- | --- |
| Minimum^3^ | 1.2 to 1.7 | 0.9 and 1.0 | -25 to -47 |
| Frontal^4^ | 2.0 to 2.4 | 1.4 and 1.7 | -30 to -42 |
| Dorsal | 2.1 to 2.6 | 3.3 and 3.4 | +30 to +62 |
| Lateral | 1.8 to 2.2 | 2.3 and 2.4 | +9 to +33 |
| Ventral^5^ | 2.2 to 2.9 | NA | NA |
| Posterior^5^ | 2.0 to 2.4 | NA | NA |

^1^We calculated the average IO angle from the lowest and highest ranges of the partial IO angles provided by Giger (1996).

^2^‘Worst case’ differences are calculated from both the low and high IO reference angles.

^3^The minima IO angle indicated by Giger (1996) is directed at 0° el. and -30° az. We found the minimum IF angles were directed towards approximately -10° el. and -65° az. for both bees in our study.

^4^We found the closest CP border to the frontal direction, which was directed at approximately -5° el. and -10° az. for both bees.

^5^We do not report ventral or posterior IF angles as the limit of the CP for the honeybees in this study did not approach those points. The posterior measurement from Giger (1996) was located at 0° el. and -140° az.
