# Supplementary material for: Bumblebee visual allometry results in locally improved resolution and globally improved sensitivity
Source: eLife. 2019 Feb 26;8:e40613. doi: 10.7554/eLife.40613 (PMC6391067; doi:10.7554/eLife.40613)
Supplement: Transparent reporting form [file elife-40613-transrepform.pdf]

eLIFE
1st Floor
24 Hills Road
Cambridge CB2 1JP, UK
P 01223 855340
W elifesciences.org
T @elife

eLife's transparent reporting form

We encourage authors to provide detailed information *within their submission* to facilitate the interpretation and replication of experiments. Authors can upload supporting documentation to indicate the use of appropriate reporting guidelines for health-related research (see EQUATOR Network), life science research (see the BioSharing Information Resource), or the ARRIVE guidelines for reporting work involving animal research. Where applicable, authors should refer to any relevant reporting standards documents in this form.

If you have any questions, please consult our Journal Policies and/or contact us: editorial@elifesciences.org.

Sample-size estimation

- You should state whether an appropriate sample size was computed when the study was being designed
- You should state the statistical method of sample size computation and any required assumptions
- If no explicit power analysis was used, you should describe how you decided what sample (replicate) size (number) to use

Please outline where this information can be found within the submission (e.g., sections or figure legends), or explain why this information doesn't apply to your submission:

We did not conduct a power analysis as this was a descriptive anatomical study.

We state in the 'Study animals' subsection of the 'Methods' that bumblebees were chosen to cover the range of sizes observed in a colony, which is sufficient to describe their eye allometry.

Replicates

- You should report how often each experiment was performed
- You should include a definition of biological versus technical replication
- The data obtained should be provided and sufficient information should be provided to indicate the number of independent biological and/or technical replicates
- If you encountered any outliers, you should describe how these were handled
- Criteria for exclusion/inclusion of data should be clearly stated
- High-throughput sequence data should be uploaded before submission, with a private link for reviewers provided (these are available from both GEO and ArrayExpress)

Please outline where this information can be found within the submission (e.g., sections or figure legends), or explain why this information doesn't apply to your submission:

eLife Sciences Publications, Ltd is a limited liability non-profit non-stock corporation incorporated in the State of Delaware, USA, with company number 5030732, and is registered in the UK with company number FC030576 and branch number BR015634 at the address 1st Floor, 24 Hills Road, Cambridge CB2 1JP | August 2014

We state in the ‘Volumetric analysis’ subsection of the ‘Methods’ that the two best preserved samples (based on qualitative inspection of our microCT images) for each size category and species were used in our analysis. These are biological replicates. Technical replication was not performed in the conventional sense. However, most variables were computed at 25 micrometer intervals across the surface of the eye as described in the ‘Computational analysis’ subsection of the ‘Methods’ – this provides both a topology and distribution of each variable for every eye.

To establish the reliability of our analysis method we analyzed the eyes of two honeybees (which are known to have highly consistent body size and eye morphology). We state in the ‘Comparison to other studies on honeybee and bumblebee eyes’ subsection of the ‘Discussion’ that the results of all computed measurements are consistent between these two individuals. Furthermore, we extensively compare our results from honeybees and medium sized bumblebees to other results published in the literature, from which we conclude that our method produces results that are accurate to within 5% of the values provided by conventional compound eye analysis techniques.

Statistical reporting

- Statistical analysis methods should be described and justified
- Raw data should be presented in figures whenever informative to do so (typically when N per group is less than 10)
- For each experiment, you should identify the statistical tests used, exact values of N, definitions of center, methods of multiple test correction, and dispersion and precision measures (e.g., mean, median, SD, SEM, confidence intervals; and, for the major substantive results, a measure of effect size (e.g., Pearson's r, Cohen's d)
- Report exact p-values wherever possible alongside the summary statistics and 95% confidence intervals. These should be reported for all key questions and not only when the p-value is less than 0.05.

Please outline where this information can be found within the submission (e.g., sections or figure legends), or explain why this information doesn't apply to your submission:

Raw data (mean values and distributions) are presented for all computed measurements for each sample, and one individual topology is shown for a small, medium, and large bumblebee, and for a honeybee.

The only statistical method performed was linear regression to calculate power functions, which is the standard for allometry studies. The functions are written beneath where they are plotted, and all parameters (R^2 and p values) for these functions are listed in Table S1.

(For large datasets, or papers with a very large number of statistical tests, you may upload a single table file with tests, Ns, etc., with reference to sections in the manuscript.)

Group allocation

- Indicate how samples were allocated into experimental groups (in the case of clinical studies, please specify allocation to treatment method); if randomization was used, please also state if restricted randomization was applied
- Indicate if masking was used during group allocation, data collection and/or data analysis

Please outline where this information can be found within the submission (e.g., sections or figure legends), or explain why this information doesn't apply to your submission:

We sampled insects from their colonies on the basis of their size and species which form the groups within this study, as stated in the 'Study animals' subsection of the 'Methods'.

Masking was not possible as the size/species were clearly apparent for the researchers performing sample collection and image analysis.

Additional data files ("source data")

- We encourage you to upload relevant additional data files, such as numerical data that are represented as a graph in a figure, or as a summary table
- Where provided, these should be in the most useful format, and they can be uploaded as "Source data" files linked to a main figure or table
- Include model definition files including the full list of parameters used
- Include code used for data analysis (e.g., R, MatLab)
- Avoid stating that data files are "available upon request"

eLife Sciences Publications, Ltd is a limited liability non-profit non-stock corporation incorporated in the State of Delaware, USA, with company number 5030732, and is registered in the UK with company number FC030576 and branch number BR015634 at the address 1st Floor, 24 Hills Road, Cambridge CB2 1JP | August 2014

Please indicate the figures or tables for which source data files have been provided:

We state in the 'Supplemental material' that we plan to upload the original data (volumes and labels) to the MorphoSource repository, and the computed measurements to the DataDryad repository, upon acceptance of this manuscript.

Ascension numbers to access these data will be included in an additional table.

We are currently preparing the extensive code base used in this study for open-source release - we hope to include a repository link in the final manuscript, and state in the 'Supplemental material' that we can currently provide the code upon request.
